# The Role of Biofilms in the Pathogenesis of Animal Bacterial Infections

**DOI:** 10.3390/microorganisms11030608

**Published:** 2023-02-28

**Authors:** Live L. Nesse, Ane Mohr Osland, Lene K. Vestby

**Affiliations:** 1 Department of Animal Health, Welfare and Food Safety, Norwegian Veterinary Institute, 1433 Ås, Norway; 2 Department of Analysis and Diagnostics, Norwegian Veterinary Institute, 1433 Ås, Norway

**Keywords:** biofilm, animals, endocarditis, meningoencephalitis, wound infections, endometritis, mastitis, respiratory disease, subclinical infections

## Abstract

Biofilms are bacterial aggregates embedded in a self-produced, protective matrix. The biofilm lifestyle offers resilience to external threats such as the immune system, antimicrobials, and other treatments. It is therefore not surprising that biofilms have been observed to be present in a number of bacterial infections. This review describes biofilm-associated bacterial infections in most body systems of husbandry animals, including fish, as well as in sport and companion animals. The biofilms have been observed in the auditory, cardiovascular, central nervous, digestive, integumentary, reproductive, respiratory, urinary, and visual system. A number of potential roles that biofilms can play in disease pathogenesis are also described. Biofilms can induce or regulate local inflammation. For some bacterial species, biofilms appear to facilitate intracellular invasion. Biofilms can also obstruct the healing process by acting as a physical barrier. The long-term protection of bacteria in biofilms can contribute to chronic subclinical infections, Furthermore, a biofilm already present may be used by other pathogens to avoid elimination by the immune system. This review shows the importance of acknowledging the role of biofilms in animal bacterial infections, as this influences both diagnostic procedures and treatment.

## 1. Introduction

**Figure 1:**
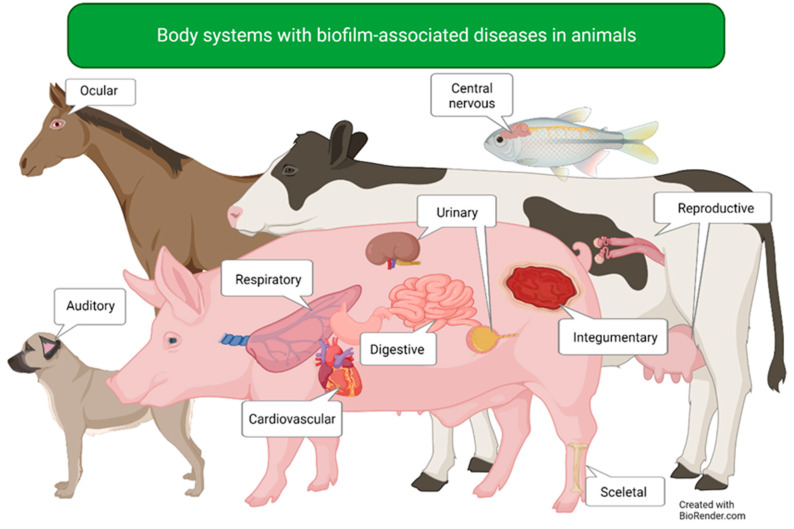
Body systems with biofilm-associated diseases addressed in this review.

In the 1970s, biofilms were observed in a *Pseudomonas aeruginosa* (*P. aeruginosa)* chronic lung infection in humans and have since then been presumed to play a part in the pathogenesis of chronic infections [1,2,3,4]. Most, if not all, bacteria may form biofilms. The clinical significance of biofilms in human disease has been investigated in several studies in recent decades. They have been found to affect numerous organ systems such as the auditory, cardiovascular, digestive, integumentary, reproductive, respiratory, and urinary systems, and this was thoroughly reviewed by Vestby et al. in 2020 [5]. However, not nearly the same amount of research has been devoted to biofilm infections in animals (Figure 1). Since the 1980s, biofilm-related infections in domestic animals have been researched, with accentuation on the infections with the largest economic burdens (e.g., mastitis, endometritis, and respiratory infections). In addition to the obvious importance for food production as well as for the individual animal, there is also a One Health aspect as infections residing in animals may be a potential danger, also to humans. Infections may have zoonotic potential, with animals as infective, possibly asymptomatic, carriers. An advantage of studying animal infections is the possibility of also including experimental infections, adding even more valuable knowledge. Concurrently, these studies may be explicitly designed and directed specifically toward a certain species. In human diseases, merely natural infections may be studied. To fully comprehend the response to in vivo biofilms, one cannot depend on in vitro experiments alone [6].

Biofilms are the most prevalent mode of life for bacterial cells and may be mono- or polymicrobial [7,8]. In biofilms, the bacteria form aggregates and are embedded in a self-produced protective matrix composed of extracellular elements such as eDNA, polysaccharides (e.g., alginate), and proteins (e.g., fibrin) [9]. Here, bacteria communicate through signaling molecules, regulated by complex quorum sensing (QS) [10], and alter their gene expression and protein production. Horizontal gene transfer may also occur in this community [11,12]. Following infection, different scenarios may occur depending on the infective agent, host immune defense, and site affected. The cells in biofilms may remain hidden from the body’s immune system due to the protection of the matrix or due to intracellular biofilm formation [13,14], which will permit chronic subclinical infections to occur. Activation of both the innate and adaptive immune systems may also ensue, leading to a longstanding inflammation, mainly with polymorphonuclear neutrophils (PMNs). This can cause tissue damage and necrosis [6] whereas the biofilm remains unaffected. As the biofilm matures and disperses [15], there is a possibility of acute infections developing or infections spreading elsewhere in the body. Moreover, the presence of a biofilm has been found to ease intracellular invasion [5]. Within the biofilm, bacteria are well protected against outside forces such as bactericides/antibiotics [12,13,16,17], and the bacteria alter their metabolism to tolerate less nutrient availability and anoxia [18], developing a tolerant subpopulation. All bacteria may acquire genetic resistance (also while in biofilms) to antibiotics; however, the tolerance appearing in the protection of biofilms is particular to this phenotype. This tolerance renders bacteria in biofilms substantially more difficult to treat than planktonic bacteria. In essence, infections involving biofilms are usually slowly developing, persistent infections where the immune system is defeated and the response to antimicrobial treatment is inconsistent. Accordingly, biofilms are likely to be essential in the pathogenesis of chronic disease.

This review aims to give a detailed description of observations and indications of biofilms in bacterial infections of diverse organ systems in animals (Figure 1) as well as the possible role biofilms may have in the pathogenesis of these infections. Biofilms on teeth and organ implants and the treatment of biofilm infections are out of the scope of this review. This review will encompass husbandry animals, such as ruminants, pigs, and fish. It will also include companion and sport animals, such as dogs, cats, and horses. Lastly, it will enclose topics on experimental animals, such as rats, mice, and rabbits that may be relevant and informative to the field.

## 2. Auditory System

### Canine Otitis Externa (OE)

OE is a frequently reported disorder in dogs, associated with infections by *Staphylococcus pseudintermedius* (*S. pseudintermedius*) and *P. aeruginosa* in addition to yeast pathogens [19]. Cytological observations of microbial aggregates and filamentous veil-like materials in clinical practice have earlier been indicative of the presence of biofilms [20]. In smears from human otitis media with effusion, periodic acid-Schiff (PAS) has been used to visualize polysaccharide biofilm matrix components.

**Figure 2:**
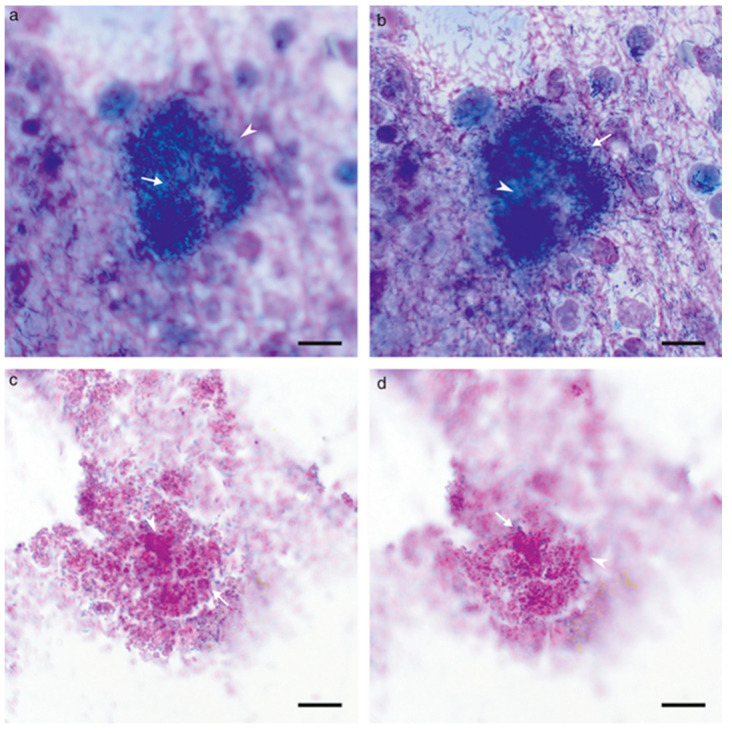
Comparison of bacterial aggregates across two focal planes from slides assessed to have aggregate-associated infection. In-focus microorganisms are indicated with white arrows; out-of-focus microorganisms indicated with the white arrowheads. Note the difference in the focus of organisms across focal planes. Microorganisms are high-density and clustered within a well-defined stained matrix, versus organisms on the periphery, which are not associated with the matrix. Both samples are from the same ear. (**a**,**b**) modified Wright’s stain, (**c**,**d**) periodic acid-Schiff stain. (**a**–**d**) 100× (oil) objective, bar = 10 μm. Reprinted from [20], Copyright (2021), with permission from John Wiley and Sons.

In a study by Parnell-Turner et al. to identify biofilms in dogs with OE, several investigators studied cytological smears from both patients and controls stained with PAS and modified Wright’s stain in a blind test [20]. The term microbial aggregate was defined as: “(i) a distinct, dense mass of apparently connected micro-organisms; and (ii) different micro-organisms that come into focus across two or more focal planes”. In addition, to meet the criteria for aggregate-associated infection (AAI): “the slide had to satisfy the criteria for presence of purulence, infection and at least one microbial aggregate present within a discrete area of stained extracellular matrix (i.e., suspected EPS)”. There was a good agreement between the investigators on the findings in PAS-stained smears, which they found easier to read than the smears stained with modified Wright’s. In the smears from the patients, the investigators could observe high-density aggregates of microorganisms within a well-defined stained matrix (Figure 2). This was not observed in any of the controls. The findings show that biofilms are associated with OE in dogs.

## 3. Cardiovascular System

Myocarditis is inflammation of the heart muscle (myocardium) [21]. The myocardium is the muscular layer of the heart and consists of cardiac muscle cells. The papillary muscles of the left ventricular myocardium are often involved in myocarditis, often visualized by purple discoloration of the overlying endocardium [22]. A study has also shown that biofilm-like aggregates of bacteria can occur in capillaries and veins in the myocardium [23]. The number of studies on myocarditis in animals is very limited.

### Bovine Myocarditis

In bovine, *Histophilus somni* (*H. somni)* has been found to be the most common causative agent in myocarditis infection that results in sudden death, and the importance of *H. somni* in this disease has been recognized since the 1980s [21,23]. *H. somni* is a non-motile, Gram-negative bacterium that is a facultative anaerobe. It belongs to the family Pasteurellacease. *H. somni* biofilms have been shown to be particularly prominent in the cardiac tissue of myocarditis cases [21].

A study by Sandal et al. [21] claimed to have found evidence that biofilms play a role in the pathogenesis of both myocarditis and bovine respiratory disease complex (BRDC) in calves (see chapter 8.1 for results on BRDC). In that study, four male calves were challenged with *H. somni*, while one calf was left unchallenged as a control. Post-mortem, myocardial necrosis was found in various degrees in the challenged calves. Histopathology showed suppurative myocarditis. A variety of advanced microscopy techniques, in combination with different staining techniques (transmission electron microscopy, immunoelectron microscopy, scanning electron microscopy, and fluorescence in situ hybridization) were used to visualize biofilm matrix components. This revealed that large amounts of biofilm matrix were present. The authors hypothesize that it is the anaerobic environment in the myocardium that results in the prominent biofilm formation in this area [21]. A study by Elswaifi et al., focusing on respiratory tract infection, suggested that one of the *H. somni* virulence factors is the presence of phosphorylcholine (ChoP) on lipooligosaccharide (LOS) occurring in the antigenic phase. This may reduce the host inflammatory response and promote the formation of stable biofilms. LOS antigenic variation may occur through variations in the composition or structure of glycoses or their substitutions, such as ChoP [24].

## 4. Central Nervous System

Knowledge is sparse regarding possible biofilm formation within the central nervous system. However, a study on tilapia fish showed biofilm formation in the brain with the fish pathogen *Streptococcus agalactiae* (*S. agalactiae*), i.e., group B streptococcus, GBS [25]. Furthermore, the presence of a biofilm was associated with the development of chronic subclinical meningoencephalitis in the fish.

### Chronic Streptococcal Meningoencephalitis in Fish

*S. agalactiae* (GBS) was first associated with bovine mastitis. It is also known to persist asymptomatically in the human digestive and genitourinary tracts, as well as in the upper airways, and it is the main cause of pneumonia, bacteremia, and meningitis in neonates [26]. Furthermore, it infects a number of fish species [27,28]. Piscine GBS (PiGBS) penetrates the intestinal mucous membrane and travels via the bloodstream to the brain where it causes chronic meningoencephalitis [29]. It has been suggested that nucleated erythrocytes facilitate brain invasion leading to granulomatous inflammation.

**Figure 3:**
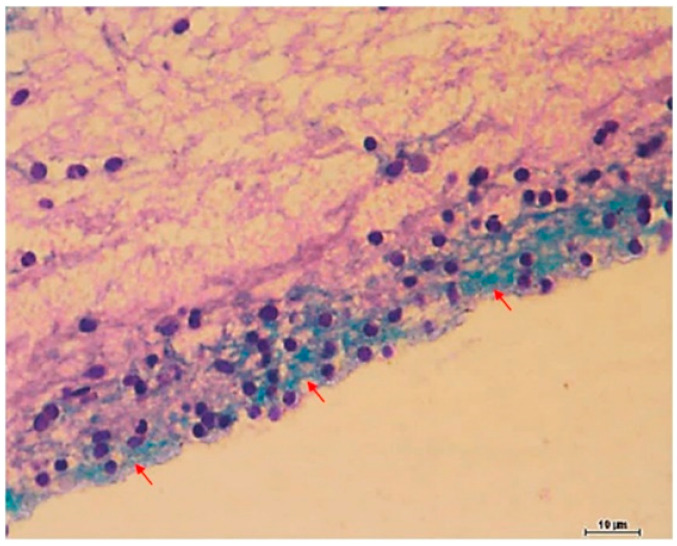
Representative image of exopolysaccharide (EPS) matrix on meninges of the tilapia model. Piscine GBS on the meningeal surface secretes acidic polysaccharides—light blue (red arrows). Neutral mucopolysaccharides of the brain parenchyma—magenta. Nucleus—dark blue. Myelencephalon, AB/PAS stain. For interpretation of the references to color in this figure legend, the reader is referred to the web version of this article. https://doi.org/10.1016/j.micpath.2016.10.029, accessed on 7 November 2022. Reprinted from [25], Copyright (2017), with permission from Elsevier.

In the study of Isiaku et al. [25], red hybrid tilapia fish were orally given PiGBS (exposed) or phosphate-buffered saline (controls). The exposed group displayed acute clinical signs. During the first nine days, 20% died and 17% were excluded due to severe lesions. Thereafter, 53% of the fish progressed into a chronic, subclinical state. In these individuals, biofilms in the form of PiGBS aggregates were observed around the meningeal surfaces and within attached substances of an exopolymeric matrix with a considerable amount of exopolysaccharides using PAS staining (Figure 3), fluorescence in situ hybridization and confocal laser scanning microscopy. These PiGBS were not eliminated by inflammatory responses in the brain, nor by treatment with antibiotics, i.e., displaying the typical feature of biofilm-residing bacteria.

## 5. Digestive System

The huge microbiota of the gastrointestinal tract consists of a large number of bacterial species moving between different lifestyles, i.e., as planktonic, biofilm-residing and biofilm-dispersed bacteria [30]. Natural polymicrobial biofilms are present throughout the gastrointestinal tract, where they are attached to mucin or food particles in the lumen or to the epithelial surface. Disease-associated biofilms may have altered bacterial and/or matrix composition, attachment, organization, metabolite production, or other abnormal features.

In humans, inflammatory bowel disease (ulcerative colitis and Crohn’s disease) and colorectal cancer have been associated with biofilms dominated by *Bacteroides fragilis* and *Enterobacteriaceae* adhering to the epithelium [5]. Gastric biofilms have been observed in *Helicobacter pylori*-positive patients, as well as in an *H. pylori* mouse model [31]. In addition, biofilms of *Salmonella enterica* serovar *Typhi* on gallstones have been indicated to cause the carrier state of this bacteria, often resulting in complications such as hepatitis, chronic diarrhea, pancreatitis, and even hepatobiliary carcinomas [5]. However, little is known about the role of biofilms in animal gastrointestinal infections.

### Salmonella Infections

Most serovars of non-typhoid *Salmonella enterica* (*S. enterica*) rarely give clinical symptoms in healthy adult animals. However, the bacteria can colonize the gut and thereby enter the human food chain, causing human outbreaks of salmonellosis. Direct or indirect contact with domesticated or wild animals may also be a source of infection for humans. A study in mice has strongly indicated that orally ingested *Salmonella enterica* serovar Typhimurium (STM) can produce an intestinal biofilm. It is probable that this may be the case in other animals as well, but the significance of such a biofilm in relation to the carrier state and/or to the development of clinical disease is still unknown.

The study was focused on an important component of the salmonella biofilm matrix [32]. This component was curli, i.e., an extracellular, bacterial amyloid, also called curli fimbriae. To investigate whether STM could produce curli in vivo, Miller et al. infected mice orally with STM grown so that curli were not present in the inoculum. After four to six days, small bacterial clusters or microcolonies were observed within the cecum. Immunohistochemistry staining confirmed that these bacterial cells were curli-producing STMs. The bacteria were also detected in the colon, where they were tightly packed, and had the most intense curli staining. On the other hand, STMs in the small intestine were sporadically spread and not curli-producing. Furthermore, SDS-PAGE investigations showed that curli was not present in the colon of control mice, confirming that the observed curli in the inoculated mice was not produced by other bacterial species in the microbiota. Mice were also inoculated with an STM luciferase reporter strain for the expression of the *csgD* gene, which encodes the major regulator of biofilm formation in STM. The expression of this gene was observed in the GI tracts of all infected mice. Together, these results indicate the production of an intestinal STM biofilm in the lower GI tract.

## 6. Integumentary System

**Figure 4:**
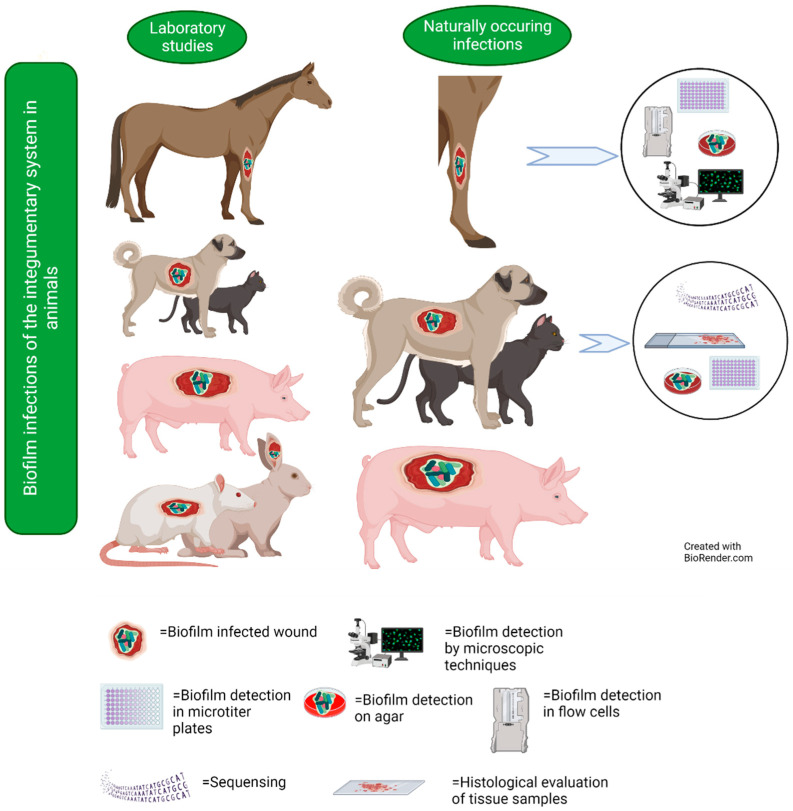
Studies on naturally occurring and experimental wound infections, including information on techniques that have been used in studies on natural infections in horses and dogs. Below are explanations of the symbols used in the figure.

Wounds occur when living tissue is damaged. Most wounds containing microorganisms do heal successfully but may result in infections if microorganisms multiply and the healing process is disrupted. This applies to both humans and animals [33]. Although it is generally accepted that biofilms are involved in many wound infections, only a few studies on naturally occurring biofilm wound infections in animals have been conducted. There is a limited number of studies on equine [34,35,36] and approximately the same number of studies on companion animals such as cats and dogs [37,38,39]. In addition, several experimental studies have been performed (Figure 4).

### 6.1. Studies on Naturally Occurring Wound Infections

As for naturally occurring wound infections in veterinary medicine, horses are at risk of developing chronic wounds on limbs, and those wounds have many similarities to chronic wounds in humans [40]. A study by Westgate et al. claims to have found evidence that as much as 61.5% (8 out of 13) of naturally occurring wounds from equines are biofilm infections. In that study, anaerobic and aerobic bacteria were isolated from the wound site, and it was found that *P. aeruginosa* and *Staphylococcus aureus* (*S. aureus*) were the two most dominating bacterial species isolated from equine wounds. When testing the biofilm-forming abilities of the isolates from the wounds versus isolates from equine skin, it was found that isolates from equine wounds formed significantly more biofilms (*p* < 0.05) in microtiter plates than isolates from equine skin [34]. However, the validity of in vitro methods as models for in vivo biofilms is disputed and especially in vitro models based on microtiter plates or flow cells. One reason for this is suggested to be due to differences in biofilm culture conditions in vitro and in vivo as in vitro conditions often involve well-defined minimal culture media, whereas in vivo conditions involve blood and other bodily fluids as the growth environment [41].

A recent review article by Jørgensen et al. concluded that *P. aeruginosa* and *S. aureus* were the two most dominant bacterial species in biofilm wound infections [33]. The same authors have, in a previous experimental study, found that biofilms were present in 100% of surgically prepared and bandaged wounds of equine limbs. In contrast, biofilms were not found in body wounds. The authors speculate that this is due to the diminished and prolonged inflammatory response detected in limb wounds compared to body wounds or the hypoxic conditions of equine limb wounds. In that study, the authors present a model for studying wound infection in horses using an equine laboratory animal model [42]. Microscopical techniques using peptide nucleic acid (PNA), fluorescence in situ hybridization, and confocal laser scanning microscopy were used in the study to categorize the wounds as biofilm-infected wounds or not [42]. These techniques have been suggested to be the gold standard for the visualization of biofilm infections in wounds in veterinary medicine [33].

A study by Swanson et al. is claiming to be the first study that reports a bacterial biofilm in chronic wounds in dogs. That study uses 16S rRNA fragment sequencing and pyrosequencing to identify microorganisms present in a pressure wound on one dog. In addition, the biofilm was claimed to be identified histologically [39]. A different study using ninety-one historical formalin-fixed and paraffin-embedded samples from dogs (n = 68), cats (n = 15), and horses (n = 8) reported to have found evidence of biofilms in only two of the samples examined. Both biofilms were found in canine tissue samples and contained exclusively cocci, but due to the nature of the samples, complete identification of the bacterial species was not possible [37]. The same authors have found in a different study that the most dominant bacterial species from wounds of dogs are staphylococci and streptococci [38]. Although the number of samples tested in the study is fairly high, the study uses criteria to determine whether or not a biofilm is present in the sample described in a study from 2003 [43], so its relevance may be disputable. None of the studies addresses the pathogenesis.

### 6.2. Laboratory Studies on Wound Infections

In contrast to the limited number of studies on naturally occurring biofilm wound infections, there are several studies that have been conducted using laboratory animals, mainly pigs [44], rabbits, and rodents [45,46], but also equines as described in the above paragraph [42]. Studies using laboratory animals are mostly used as models with the purpose of better understanding infections in humans [47] but may also be used as models for a better understanding of infections in animals [42]. Wound healing in rodents happens by contraction in contrast to epithelialization and granulation in humans [45]. Using a rodent model to study biofilms and wounds showed that polymicrobial biofilms delay wound closure and healing [48,49]. Using a rabbit ear model, biofilms were found to significantly delay epithelialization and granulation tissue formation. Wounds containing biofilms were found to significantly express lower levels of inflammatory cytokines than infected wounds [50]. Another study found that biofilms were formed in ischemic wounds but not in non-ischemic wounds where neutrophils and macrophages were present [51]. The wound healing process in pigs occurs through inflammation, proliferation, re-epithelization, and remodeling, which is analogous to humans [45]. Pastar et al. found that using a partial thickness wound model in pigs, the delayed healing of wounds was found due to the suppression of epithelialization and the expression of virulence factors [52].

## 7. Reproductive System

### 7.1. Endometritis

Successful reproduction is essential in animal husbandry, and failures cause vast economic expenses. Research on diseases of the reproductive system in animals is therefore of major interest. Endometritis is the inflammation of the lining of the uterus, usually involving infection. The uterine environment has been assumed to be sterile, but recent studies in humans [53] and several animal species [54,55,56,57], have revealed the presence of a uterine microbiota. Uterine biofilms have also been observed in humans [58], as well as in animals. The significance of biofilms in the pathogenesis of endometritis has been demonstrated in relation to the pyometra form of endometritis in dogs. In other animal species and in humans, the role of biofilms in endometritis has still not been clarified.

#### 7.1.1. Endometritis in Dogs

Pyometra is a potentially life-threatening, suppurative bacterial endometritis with the accumulation of inflammatory exudate [59]. It is one of the most common diseases of adult nulliparous female dogs. *Escherichia coli* (*E. coli*)*,* which is most often isolated, may produce endotoxin resulting in sepsis, shock, and renal failure. Other species of the microbiota of the gastrointestinal and genitourinary tract of dogs, e.g., *S. intermedius* and β-hemolytic streptococci, have also been associated with the disease.

Fiamengo et al. found biofilms associated with the disease when examining endometrial biopsies from 16 bitches with pyometra positive for *E. coli* [60]. Histopathology from all cases showed suppurative inflammation, as well as surface and luminal exudate. Chronic inflammation was observed in 15 cases by the presence of lymphocytes and plasma cells associated with cystic hyperplasia. In 14 of the 16 cases, histopathology identified superficial basophilic amorphous acellular material, which displayed a bright pink color with PAS staining. Furthermore, fluorescence in situ hybridization techniques identified *E. coli* being present within this material. In addition, a biofilm was indicated by scanning electron microscopy observations of the fibrous matrix on the luminal surface of the endometrium in all samples. Neither the bacteria nor the matrix was observed in control samples from two healthy bitches.

#### 7.1.2. Endometritis in Mares

Endometritis is a primary cause of infertility in mares, contributing to major economic losses. It is generally caused by post-breeding failure to remove spermatozoa, inflammatory exudate, and bacteria [61]. *Streptococcus equi* subsp. *zooepidemicus, E. coli*, *Klebsiella pneumoniae*, and *P. aeruginosa* are commonly identified. Endometritis can be difficult to treat with antibiotics, a feature that has suggested intrauterine biofilm production to play a role [62].

**Figure 5:**
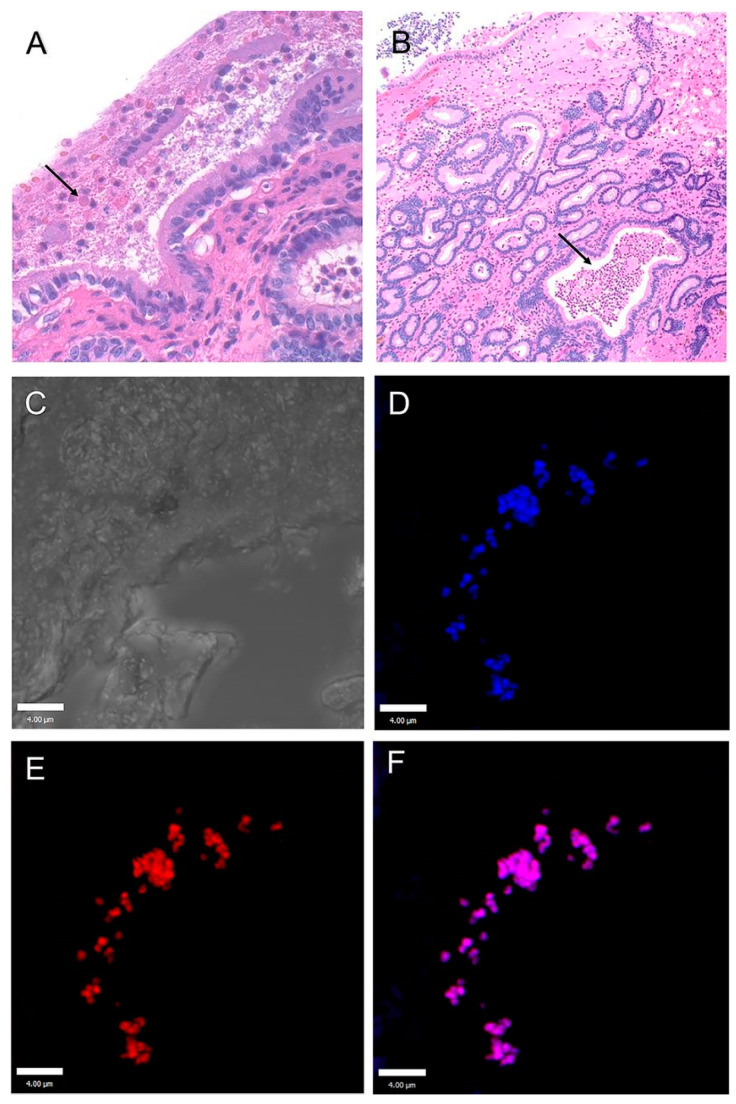
Detection of tissue-adherent *P. aeruginosa* in endometrium samples. H&E image of the endometrium with tissue-adherent *P. aeruginosa* on the luminal surface (black arrow) (**A**) and deep in the endometrial glands (black arrow) (**B**). (**C**) Differential interference contrast image of an endometrial gland below the luminal surface of the uterus; this is similar to the area represented in panel B by the black arrow. Immunofluorescent staining of tissue-adherent *P. aeruginosa* with an anti-*Pseudomonas* antibody (Alexa Fluor 405) (**D**) and anti-Pel lectin (Texas red) (**E**) and merged image detecting the Pel exopolysaccharide colocalized with *P. aeruginosa* (**F**). Immunofluorescent images are projected images of Z-stacks as processed by Volocity image analysis software in which 0.5-μm scanning increments were performed through approximately 10 μm of tissue. The scale bar is 4 μm. Reprinted from: Ferris et al. 2017 [64].

Indeed, *P. aeruginosa* has been shown to form biofilms on the endometrial surface of mares. Ferris et al. [63] developed an in vivo model to monitor intrauterine biofilms in infectious endometritis, in which a mare was inoculated with three *lux*-engineered *P. aeruginosa* strains isolated from equine uterine infections. Five days later, an ultrasound revealed excessive edema and inflammation. When opening the uterus, the presence of a biofilm was indicated by observations of a luminescent, strongly adherent material, which was positive for *P. aeruginosa* and was observed on the endometrial surface. The model was further used to study the spatial intrauterine localization of metabolically active bacteria in six mares [64]. The biofilm matrix component Pel was observed in endometrial samples with tissue-adherent bacteria (Figure 5). Interestingly, reduced numbers of neutrophils, as well as the increased gene expression of the immune-modulatory, anti-inflammatory Interleukin 10, was observed in areas surrounding tissue-adherent bacteria. This indicates a modulated immune response in such areas. However, tissue inflammation was the same in areas with and without biofilm.

### 7.2. Endometritis in Cows

Postpartum endometritis is one of the main causes of reduced fertility in dairy cattle. Metagenomic analyses have shown that the uterine microbiota of cows with clinical endometritis displays an increased abundance of *Fusobacterium* and a unique presence of *Trueperella* and *Peptoniphilus* [65]. The increased prevalence of *Trueperella pyogenes* (*T. pyogenes*), earlier named *Arcanobacterium pyogenes*, in the microbiome of cows with endometritis, has also been reported in other studies [66,67].

Amaci et al. [68] investigated secretion samples from 20 repeat breeder cows, i.e., cows who failed to conceive after three or more inseminations with fertile semen without any anatomic or infectious abnormality. The smears were prepared from the sediment of centrifuged uterine saline lavage. Biofilms visualized as red complexes with PAS staining were observed in 12 animals (60%). Bacteria were isolated from eight animals, all of them displaying biofilms in the smears. There was no correlation between the presence of a biofilm and the results from the cytological examinations of uterine secretion. Furthermore, there was no control group in this study. Consequently, the study demonstrated the presence of an intrauterine biofilm in repeat breeder cows, but a possible effect on fertility or the development of endometritis is unknown.

Interestingly, the commonly found bacterium related to endometritis in cows, *T. pyogenes*, has a number of adherence mechanisms, including several extracellular matrix-binding proteins and fimbriae [69]. Furthermore, biofilm formation is upregulated by the same two-component regulatory system that upregulates the expression of the major virulence factor pyolisin. Although this may indicate that *T. pyogenes* is pathogenic when residing in biofilm, it is presently not known whether biofilms are in fact a part of the pathogenesis of endometritis.

### 7.3. Mastitis

Mastitis is the inflammation of the mammary gland. It is one of the most challenging diseases in the dairy industry, causing enormous economic losses. This has prompted a large interest in bovine and small ruminant mastitis research in general and a possible role of biofilms in particular. However, the majority of the latter studies have been restricted to in vitro investigations on the biofilm-forming abilities of clinical isolates. Relatively few studies have focused on direct or indirect observations of biofilms’ presence in the udders of affected animals in vivo. Even so, these few studies indicate both the presence of biofilms and a possible role in the pathogenesis of mastitis.

#### Mastitis in Cows

Bovine mastitis occurs primarily during lactation. The main pathogen is S. aureus. Other mastitis-associated bacteria are *Streptococcus agalactiae* (causing contagious mastitis), coagulase-negative staphylococci (CoNS), *E. coli*, *Klebsiella* spp., *Enterobacter* spp., *Citrobacter* spp., *S. dysgalactiae*, *S. uberis*, *Enterococcus* spp., and *Pseudomonas* spp.

Observations indicating presence of biofilms were made by Hensen et al. when they experimentally infected two quarters in each of three lactating cows with low doses of S. aureus [70]. The cows were slaughtered at different time points, and the udders were subjected to macro- and microscopic examinations. During the first four days after inoculation, all cows displayed mild clinical mastitis in the infected quarters, and two of the cows were slaughtered. When the third cow was slaughtered after 122 days, it showed signs of chronic subclinical mastitis with high somatic cell counts in the milk and diffuse changes related to fibrosis in the inoculated quarters. In tissue samples from the cows in both the clinical and chronic subclinical stages, Gram-positive cocci were mainly located in clusters in the alveoli and lactiferous ducts in association with inflammatory cells. In the chronic stage, the bacteria were associated with the epithelium and were also observed within the interstitial tissue.

In another study, Schönborn and Krömker investigated swabs from slaughtered *S. aureus*-infected dairy cows [71]. Immunofluorescence staining identified polysaccharide intercellular adhesion PIA in 71 of the 184 samples. As PIA is a component of the biofilm matrix of *S. aureus*, such findings are indicative of *S. aureus* biofilms.

Vaccination studies also indicate a possible contribution by biofilms to the pathogenesis of mastitis. Gogoi-Tiwari et al. used a mouse model to study the effect of immunization with formalin-killed *S. aureus* from biofilms versus formalin-killed planktonic bacteria. The mice immunized with biofilm-residing bacteria displayed significantly lower *S. aureus* colonization, less severe clinical symptoms, and less tissue damage in their mammary glands [72]. In a study on sheep by Perez et al., vaccination with crude bacterial extracts from strong *S. aureus* biofilm formers displayed the highest production of antibodies and the best protection against infection and mastitis after intra-mammary challenge, as compared to immunization with extracts from weak biofilm formers and controls [73].

Studies on the expression of the biofilm-associated surface protein Bap in staphylococci [74] may provide even more information on a possible role for biofilms in mastitis pathogenesis. *S. aureus* bap-positive isolates from sub-clinical mastitis were found to be better to colonize and persist in the mammary gland in vivo. Furthermore, antibodies against Bap were observed in the serum of these animals, thus confirming that Bap, and thereby probably also a biofilm, was produced during the infection. Zuniga et al. [75] found that medium somatic cell counts in the milk (i.e., markers of inflammation) were higher in milk samples from sub-clinical mastitis with bap-positive staphylococci than in samples with bap-negative staphylococci. These results indicate that biofilms may contribute to the inflammatory magnitude in the udder.

Intracellular live *S. aureus* have been observed in alveolar cells in milk samples from chronic bovine mastitis [76]. Interestingly, a three-year follow-up of a dairy herd showed that strains of the most prevalent and persistent *S. aureus* genotype displayed both high biofilm production and high cellular invasiveness [77]. A similar link between biofilms and invasiveness was also observed in a study on human patients with chronic rhinosinusitis, in which the location of intracellular *S. aureus* was associated with surface biofilms [78]. This indicates that epithelial-associated biofilms may facilitate cellular invasion by *S. aureus*.

## 8. Respiratory System

Biofilms have been observed or indicated to play a role in the pathogenesis of a number of bacterial infections in the human respiratory system, e.g., chronic rhinosinusitis, pharyngitis and laryngitis, pertussis, and cystic fibrosis [5]. Most of the bacterial pathogens involved also infect animals. It is therefore not surprising that experimental studies show that the same pathogens can also produce biofilms in the respiratory systems of animals. As an example, *S. aureus*, which was inoculated in sheep sinuses, produced a biofilm identifiable by confocal laser scanning microscopy, scanning electron microscopy, and transmission electron microscopy [79]. In addition, biofilms have also been observed as being present in animal respiratory diseases.

### 8.1. Bovine Respiratory Disease Complex (BRDC)

Bovine respiratory disease complex (BRDC) causes substantial economic losses to the beef industry. Polymicrobial infections are common, with *H. somni, Pasteurella multocida* (*P. multocida*), and *Mannheimia haemolytica* being predominant bacterial species, in addition to *Mycoplasma bovis* and several viruses. Pulmonary biofilms have been observed in calves with BRDC after being experimentally challenged with *H. somni.*

**Figure 6:**
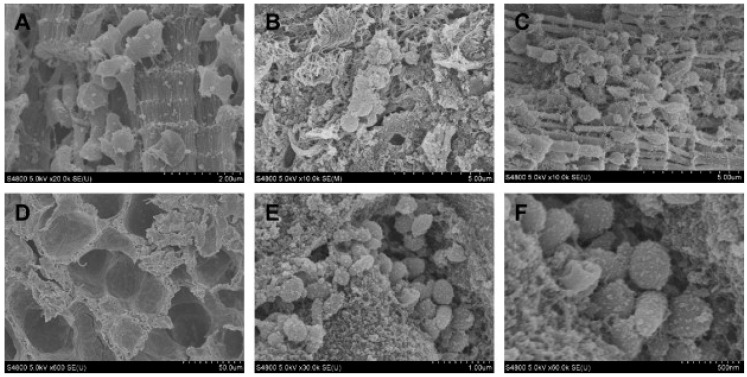
Scanning electron microscopy of freeze-fractured samples of cardiac (see chapter 3) and lung tissues. Top panel: (**A**) Scanning electron microscopy images of normal cardiac tissue; (**B**,**C**) infected cardiac tissue containing coccus-shaped bacteria surrounded in a matrix network. Scale bars: (**A**) 2 μm with magnification 20,000×; (**B**,**C**) 5 μm with magnification 10,000×. Bottom panel: (**D**) normal lung tissue; (**E**,**F**) infected lung tissue containing few coccobacilli within an extracellular matrix. Scale bars: (**E**) 1 μm with magnification 30,000×; (**F**) 0.5 μm with magnification 60,000×. Reprinted from [21], Copyright (2009), with permission from Elsevier.

Sandal et al. [21] exposed four calves to transtracheal challenge with an *H. somni* strain after four days of immunosuppressive treatment. One calf was inoculated with saline and kept as a control. All the exposed calves developed fever, respiratory symptoms, and increased respiratory and heart rates. *H. somni* was recovered from nasal swabs and transtracheal washes from all four challenged calves and *P. multocida* from three of them. The animals were euthanized after 7 days. A post-mortem examination showed suppurative bronchopneumonia. Transmission electron microscopy, immunoelectron microscopy, scanning electron microscopy (Figure 6), and fluorescence in situ hybridization investigations showed that an *H. somni* biofilm was present. Investigations of tissue from the control calf did not display such biofilms or any of the lesions observed in the infected calves. The results indicate that biofilm production is part of the pathogenesis of this disease.

Interestingly, *P. multocida* was observed in transtracheal washes and pulmonary tissue of the calves even though they had only been challenged with *H. somni*. Similar was observed in another experiment with two calves challenged with *H. somni* [80]. In these animals, both *H. somni* and *P. multocida* were present in the lungs. Furthermore, the calves produced higher antibody titers against the biofilm form than the planktonic form of both bacteria. However, only matrix components of *H. somni* were detected in the lungs. These findings taken together suggest that *P. multocida* may have entered and used the *H. somni* biofilm for persistence during chronic BRD.

### 8.2. Porcine Enzootic Pneumonia

Porcine enzootic pneumonia is a globally spread disease, which is caused by *Mycoplasma hyopneumoniae* (*M. hyopneumoniae*) and exacerbated by secondary infections and environmental stressors [81]. It usually affects growing and fattening pigs and may last for many weeks. Although the mortality is low, the morbidity is high with the pigs displaying respiratory symptoms, reduced feed efficiency, and growth retardation. This can have devastating economic consequences. Carrier pigs are responsible for persistent infections on the farm.

*M. hyopneumoniae* adheres to the ciliated respiratory epithelium, resulting in cilial loss and epithelial cell death. Early observations of the bacteria on the epithelium in experimentally infected pigs showed that they were enveloped by a thick, dark layer of capsular material. Furthermore, they had many fibrils on the surface which seemed to connect the bacteria to each other, as well to the cells [82]. Although these and other similar observations could have indicated the presence of biofilms, it took almost 40 years before biofilms were actually demonstrated in infected pigs.

Raymond et al. [83] examined tracheal sections of pigs experimentally or naturally infected with *M. hyopneumoniae*. Immunohistochemistry microscopy showed that the bacteria were located at the ciliary border of the epithelium, and aggregates of staining that resembled biofilms were observed in chronically infected pigs. Using scanning electron microscopy on samples six weeks after the experimental infection, large biofilms (100–150 µm in diameter) were observed on the epithelium. The epithelium was deciliated. Biofilms were also observed in vitro on porcine kidney epithelial-like monolayers.

### 8.3. Porcine Pleuropneumonia

Porcine pleuropneumonia is caused by *Actinobacillus pleuropneumoniae* (*A. pleuropneumoniae*). The disease, which is highly contagious and widespread, is characterized by high morbidity as well as high mortality leading to significant economic losses.

The presence of biofilms was indicated by studies of lungs from two naturally infected pigs displaying clinical signs consistent with acute porcine pleuropneumonia [84]. A microscopic examination of the lungs confirmed the clinical diagnosis by displaying typical, multiple foci of coagulation necrosis in the pulmonary parenchyma with microcolonies of small Gram-negative bacilli present. The isolation and characterization of these bacteria from the two pigs showed that they were *A. pleuropneumoniae* of serotype 7 and serotype 5, respectively, i.e., two of the most prevalent serotypes in North America. Through the use of fluorescence in situ hybridization with a species-specific oligonucleotide probe, the bacteria were observed growing as aggregates (~30–45 μm) in the lungs of both pigs, i.e., in the same way as in earlier descriptions of in vivo biofilms in human chronic lung infections [6,41]. The bacteria also formed similar aggregates in an in vitro agarose model mimicking the porous conditions of the infected loci in the lungs. The fluorescent staining of these aggregates showed the presence of poly-*N*-acetylglucosamine, which is a known component of the *A. pleuropneumoniae* biofilm matrix. These findings support the notion of the aggregates observed in vivo being biofilms.

### 8.4. Bordetella Bronchiseptica Infections

*Bordetella bronchiseptica (B. bronchiseptica)* infections have been associated with respiratory disease in a number of mammals [85]. It is widespread in swine populations and causes highly contagious tracheobronchitis in dogs and cats. In addition, long-term to life-long asymptomatic carriers shed the bacteria and infect susceptible individuals. This is most probably due to the presence of chronic, asymptomatic colonization in the form of a biofilm in the upper respiratory tract, as demonstrated in experimental infections in laboratory animals [85].

**Figure 7:**
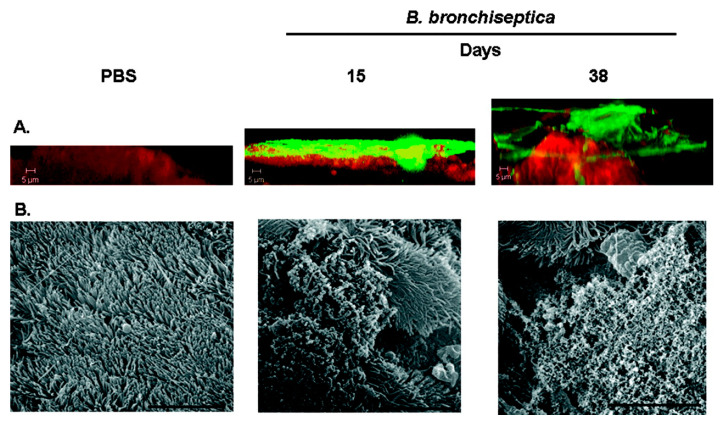
(**A**) Confocal laser scanning microscopy (CLSM) of biofilms formed within the murine nasal cavity by *B. bronchiseptica*. C57BL/6 mice were inoculated with either PBS or *B. bronchiseptica* RB50. Nasal septa were harvested at 15 or 38 days postinoculation, immediately fixed, and probed with rat anti-Bordetella serum followed by a secondary anti-rat antibody conjugated to Alexa Fluor 488 (which stains bacteria green). To determine the localization of the host epithelium, specimens were stained for F-actin using phalloidin conjugated to Alexa Fluor 633 (which stains the epithelium red) and visualized with. Each micrograph represents a Z reconstruction. For each specimen, images were obtained from at least five areas of the nasal septum and from at least three independent animals. (**B**) Scanning electron microscopy of B. bronchiseptica biofilm formation on nasal septa. Specimens were collected from animals either 15 days or 38 days post-inoculation, directly fixed, and processed for scanning electron microscopy. Scale bars = 10 μm. Reprinted from [86].

*B. bronchiseptica* biofilms in vivo have been observed in intranasal mouse models. Sloan et al. observed both bacteria and the biofilm matrix component Bps polysaccharide on the nasal septum of inoculated mice when using confocal laser scanning microscopy [86] (Figure 7A). Furthermore, scanning electron microscopy showed a densely packed multicellular community of bacteria on the ciliated epithelium with architectural features characteristic of many biofilms, i.e., mats, towers, or pillars (Figure 7B). Inoculation with a knockout mutant lacking the essential biofilm component Bps showed that this component was needed for the efficient long-term survival of *B. bronchiseptica* in the respiratory tract. No biofilms were observed in control mice. A biofilm on the nasal septa of mice after exposure to *B. bronchiseptica* was also observed by Conover et al. [87]. Confocal laser scanning microscopy showed *B. bronchiseptica* being present in scattered mat-like structures on the nasal epithelia. Scanning electron microscopy revealed that thick mats of encased bacteria in a matrix material were covering the underlying ciliated epithelium.

## 9. Skeletal System

Osteomyelitis in animals is caused by traumatic, surgical, or hematogenous infection [88]. The latter occurs more often in juveniles than in adults. Staphylococci, streptococci, *E. coli*, and other Gram-negative bacteria are often found in osteomyelitis in horses, swine, broilers, turkeys, dogs, and cats. *Erysipelothrix rhusiopathiae* is in addition found in swine, whereas *T. pyogenes* is more common in cattle. Osteomyelitis is difficult to treat, often requiring an extended duration of antibiotic therapy and repeated surgical revisions of affected tissue, i.e., hallmarks of a biofilm infection.

A number of animal models using pigs, dogs, chickens, sheep, goats, rabbits, and mice have been designed to study osteomyelitis [89,90]. Johansen et al. [91] inoculated three groups of pigs of three animals each with *S. aureus* in the right femoral artery. Group B received a porcine strain, whereas groups C and D were inoculated with two different strains of human origin. Two animals inoculated with saline were kept as controls (group A). Five days after inoculation, the pigs in group B displayed lameness of the right limb, whereas none of the others showed clinical symptoms. All animals were killed after eleven or fifteen days. Osteomyelitic lesions were observed in the right hind leg of all three pigs in group B and one in group C. These animals also harbored *S. aureus* in the femoral abscesses, as identified with PNA (peptide nucleic acid) fluorescence in situ hybridization. The bacteria were shown to reside in biofilms as aggregates of loosely-packed cocci embedded in an opaque matrix.

## 10. Urinary System

### Urinary Tract Infections

As in humans, *E.coli* is the pathogen that is known to most frequently colonize the urinary tract causing urinary tract infections (UTIs) in animals, but also Enterococcus species are frequently found. Most studies on UTIs in animals have been conducted on companion (cats and dogs) and sporting animals (horses) [92,93,94]. Studies conducted on UTIs in dogs have shown that especially female dogs are at risk. Uncomplicated canine UTIs are common and have been reported to occur in 14–17% of all dogs at one time in their lifetime. It has also been suggested that 4.5–20% of all dogs with UTIs have a recurrence of persistent UTIs [95,96]. Bacterial UTIs have been found to occur less frequently in cats than in dogs with only 1–2% of cats suffering from UTIs during their lifetime [97].

The pathogenesis of UTIs is multifactorial and depends on the interplay between bacterial virulence factors and host defense systems. A UTI can develop when the host defense mechanism is temporarily or permanently affected. Most host defense mechanisms against bacterial establishment include, e.g., the presence of normal resident microflora and physical urinary tract anatomy. Bacterial virulence factors enable bacteria to colonize and invade the urinary tract [97,98].

*E. coli* that cause infection outside the intestinal tract are called extraintestinal *E. coli* (ExPEC), and the ExPECs that colonize the urinary tract are commonly referred to as uropathogenic *E. coli* (UPEC). Experiments using intraurethral catheterization to infect mice with UPEC isolates have shown intracellular invasion and the development of structures with typical biofilm characteristics in the bladder epithelial cells (reviewed by [5]). In addition, such intracellular bacterial communities have been observed by electron microscopy in epithelial cell shedding in urine from women with UTIs.

When studying UPEC isolated from dogs, Ballash et al. found that UTI recurrence was associated with the ability to form biofilms. The study used 104 different UPEC isolates originating from 94 different dogs. The isolates were tested for their biofilm-forming capabilities in vitro using the microtiter plate method and crystal violet staining of the biofilm mass. As much as approximately 80% of the isolates were able to form biofilms in microtiter plates, where approximately 56% formed weak biofilms and 24% formed moderate or strong biofilms. Based on retrospective medical record analysis, it was found that isolates from dogs with a history of chronic/recurring urinary tract infections had 8.5 times the odds of forming a biofilm compared to isolates from dogs without a history of chronic UTIs. The same study also found that isolates that could form biofilms had a slightly higher likelihood of having more virulence genes than non-biofilm producers (*p* = 0.094) [96]. There are a few other studies using similar methodologies that also confirm that *E. coli* isolated from UTIs from dogs are able to form biofilms [99,100]. These results indicate that intracellular biofilm formation in epithelial cells also may occur during UTIs in dogs.

## 11. Visual System

### Equine Recurrent Uveitis

Equine recurrent uveitis (ERU), also known as moon blindness, is the most common cause of equine blindness with a prevalence of 2–25% worldwide [101]. ERU has been believed to be an autoimmune syndrome initiated by an episode of acute uveitis disruption of the blood–ocular barrier. However, *Leptospira* spp., which is the most common initiator, has also been found present after several episodes. Furthermore, vitrectomy, which is a treatment option for severe ERU, is most beneficial when leptospires is implicated. It is therefore not surprising that a recent study identified *Leptospira* spp. biofilms in vitrectomy samples from horses with ERU but not in samples from horses without this disease,

Brandes et al. [102] examined vitreous samples obtained by the vitrectomy of 17 horses with ERU. High titers of antibodies against leptospires were found in 16 of the 17 samples, and PCR was positive for these bacteria in all samples. This indicated that ERU can be a chronic *Leptospira* infection and that the immune response is not sufficient to eradicate the bacteria. Interestingly, an unknown homogeneous granular layer surrounded the few leptospires that were observed by using transmission electron microscopy.

Based on these and other observations, Ackermann et al. [103] suggested that the *Leptospira* biofilm might be part of the pathogenesis of ERU. To test this hypothesis, they investigated vitreous samples from 29 horses with clinical ECU and 3 horses without this clinical diagnosis. Antibodies against leptospires were found in 28 of the 29 samples from the horses with ERU. Furthermore, all 29 samples were PCR-positive for *Leptospira* spp. The samples from the control horses were negative in both tests. Microscopical investigations with Warthin-Starry Silver Stain and immunohistochemistry identified leptospires displaying biofilm formation in all ERU samples. Laboratory-cultured WHO *Leptospira* spp. strains spread on microscope slides were used as non-biofilm controls in these investigations. Three steps of biofilm formation were observed in the positive samples. Step 1: Individual leptospires surrounded by a partially granulated matrix. The bacteria displayed increased thickness as compared to the non-biofilm control leptospires. Step 2: Microcolonies consisting of bacterial aggregates in a granular matrix. Step 3: Condensed round structures with a diameter of approximately 5 to 20 µm. It was difficult to distinguish bacteria and matrix, but individual leptospires protruding from the aggregates were occasionally observed. No signs of biofilm formation could be seen in the samples from the three horses without ERU.

## 12. Discussion

Already 50 years ago, Nils Høiby observed a link between the presence of bacterial aggregates and the etiology of cystic fibrosis patients [4]. A decade later, Bill Costerton introduced the term biofilm into medicine. Since then, a large number of studies describing biofilms in relation to infection and disease in humans, as well as in animals, have been published. However, the awareness of biofilm infections among both veterinarians and physicians, in general, has been disappointingly low. This has probably affected the diagnostics and treatment of such infections.

In this review, we describe a number of observations and/or indications of bacterial biofilms in most body systems of husbandry animals, including fish, as well as in sport and companion animals (Table 1). The animals were naturally or experimentally infected. For some infections, other model animals, e.g., mice, have been used to further elucidate the formation and role of biofilms. The biofilms have been visualized by various microscopy techniques in biopsies, autopsies, and exudates. This is in compliance with the guideline from the European Society of Clinical Microbiology and Infectious Diseases (ESCMID) on the diagnosis and treatment of biofilm infections [104]. This guideline states that microscopic techniques such as confocal laser scanning microscopy and scanning electron microscopy are the most appropriate to identify biofilms in tissue samples, but light microscopy and routine staining methods may also be used. Furthermore, several of the studies described in this review have included investigations on the host’s immune response, vaccination experiments, and/or characterization of bacterial phenotypes during infection to support the observations of biofilms being present in the affected body systems. A large number of studies published have focused on measuring biofilm production in vitro by pathogens isolated from diseased animals and affected organs. Comparisons with isolates from other sources are often included. Whereas results from such studies may support observations from in vivo studies, they are themselves not sufficient to establish a link between biofilms and disease. Consequently, few such studies are included in this review.

Biofilm infections in animals can be studied under controlled conditions in experiments using the relevant animal species. The animals can be euthanized and proper organ samples collected for macro- and microscopic studies. This is in contrast to studies in humans, which are restricted to natural infections and available samples. Despite these advantages in animal studies, surprisingly few of them have looked at the possible role of biofilms in the pathogenesis of the infection studied. However, as in studies on biofilm infections in humans [5], a number of potential roles in disease pathogenesis are indicated by the observations in animal studies. Biofilms in relevant body systems of diseased animals, but not in controls, give reason to believe that biofilms are involved in the pathogenesis of clinical and subclinical infections. Biofilms can contribute to high local concentrations of bacteria. The bacteria, their metabolites, or other biofilm components may then induce the local inflammation that has been observed related to the biofilm, and that can cause or aggravate tissue damage, e.g., the deciliation of epithelium observed in porcine enzootic pneumonia. Interestingly, signs indicating that biofilms can modulate local inflammatory responses have also been observed, e.g., in equine endometritis where the increased production of anti-inflammatory Interleukin 10 was found in areas surrounding the biofilms. Furthermore, biofilms formed by one bacterial species might be used by other pathogens to avoid elimination by the immune system, as may be the case with *P. multocida* entering and using the *H. somni* biofilm for persistence during chronic bovine respiratory disease complex (BRDC). Biofilms can also act as physical barriers obstructing the healing process, e.g., in wounds. As the biofilm lifestyle offers long-term protection against both the immune system and antimicrobial treatment, it is not surprising that biofilms play a role in chronic subclinical infections, as observed in mastitis in cattle and meningitis in tilapia fish. In human studies, biofilms appear to facilitate the intracellular invasion of the underlying tissue of some bacterial species, e.g., *S. aureus*. This is supported by studies on *S. aureus* from mastitis in cows. Furthermore, studies on humans have also revealed the formation of intracellular biofilms [5], which probably increases the intracellular survival of the bacteria. This can be expected to occur in animals as well. Likewise, the observed link between biofilms and certain cancer forms in humans is probably also present in some form in animals.

Biofilm-associated infections in animals are also a cause of concern in a One Health concept. Several of the bacteria that have been found in biofilms in animals are zoonotic and may cause disease in humans, e.g., *B. bronchiseptica*, *S. aureus*, pathogenic *E. coli*, *Salmonella* spp., *K. pneumoniae*, and *P. aeruginosa.* As the biofilm formation of such pathogens promotes long-term colonization in animals, it poses an increased risk of the transfer of pathogens to humans. This can be through direct contact with animals or indirectly through contaminated food products or environments. Jacques and Malouin have therefore proposed the One Biofilm concept within the One Health concept, “where biofilm/aggregate formation in humans, animals and the environment are also intricately linked” [105].

In conclusion, it is important to acknowledge that biofilms are involved in a number of bacterial infections in animals and that this influences the choice of diagnostic procedures and treatment of these infections. Although the knowledge and awareness of the general practitioner have improved in recent years, they still need to be higher. Additionally, further development of diagnostic criteria as well as effective treatments for such infections are required. Finally, knowledge of the role of biofilms in the pathogenesis of such infections should be expanded by more studies specifically designed to address this question.

## Figures and Tables

**Table 1:** Biofilm-associated infections of different body systems and their affected organs.

Body System	Affected Organs	Disease
Auditory	Ear	Canine otitis externa
Cardiovascular	Heart	Bovine myocarditis
Central nervous	Brain	Piscine meningoencephalitis
Digestive	Intestines	Salmonella infection
Integumentary	Skin and underlying tissue	Wound infections
Reproductive	Uterus	Endometritis
	Mammary glands	Mastitis
Respiratory	Lower airways	Bovine respiratory disease
	Lower airways	Porcine enzootic pneumonia
	Lower airways	Porcine pleuropneumonia
	Upper and lower airways	*Bordetella bronchiseptica* infection
Skeletal	Bones	Osteomyelitis
Urinary	Urinary tract	Uropathogenic *E. coli* infection
Visual	Eye	Equine recurrent uveitis

## Data Availability

Not applicable.
